# Combined Metabolome and Transcriptome Analyses Reveals Anthocyanin Biosynthesis Profiles Between Purple and White Potatoes

**DOI:** 10.3390/ijms252312884

**Published:** 2024-11-29

**Authors:** Miaomiao He, Xinping Ma, Yun Zhou, Fang Wang, Guonan Fang, Jian Wang

**Affiliations:** 1 Academy of Agriculture and Forestry Sciences, Qinghai University, Xining 810016, China; hemm0505@126.com (M.H.); mxp1592@163.com (X.M.); zhouyun75@163.com (Y.Z.); qhwf324@163.com (F.W.); 17755203950@163.com (G.F.); 2 Key Laboratory of Potato Breeding of Qinghai Province, Xining 810016, China; 3 Key Laboratory of Qinghai-Tibetan Plateau Biotechnology (Qinghai University), Ministry of Education, Xining 810016, China; 4 State Key Laboratory of Plateau Ecology and Agriculture, Qinghai University, Xining 810016, China; 5 Engineering Research Center of Potato in Northwest Region, Ministry of Education, Xining 810016, China

**Keywords:** potato, anthocyanin, metabolome, transcriptome, *St5GT*

## Abstract

Colored potatoes with red and purple skin or flesh possess significant nutritional value and health benefits due to their rich anthocyanin content. To investigate the genetic mechanisms underlying color formation, the high-anthocyanin-content purple-skinned and purple-fleshed potato line 15-12-16, and the white-skinned and white-fleshed Xiazhai 65 variety were used for ultra-performance liquid chromatography-electrospray ionization-tandem mass spectrometry (UPLC-ESI-MS/MS) analysis, which was conducted to identify and quantify anthocyanins. RNA sequencing was performed to analyze the transcriptome. The results indicated a significant upregulation of genes within the anthocyanidin biosynthesis pathway in the purple potato, while these genes were either downregulated or absent in the white potato. The bHLH, MYB, and WRKY gene families exhibited a greater number of regulatory members, suggesting their pivotal role in color formation. Integrated analysis of the transcriptional and metabolic revealed that 12 differentially expressed genes (DEGs) related to the anthocyanidin biosynthetic had a significant correlation with 18 anthocyanin metabolites. Notably, the key gene *St5GT* in the anthocyanidin biosynthesis pathway was markedly upregulated in the purple skin and flesh. Furthermore, the overexpression of *St5GT* (PGSC0003DMG400004573) in tobacco contributed to anthocyanin accumulation. The expression of 10 DEGs was validated through quantitative real-time PCR. In conclusion, these findings provide new insights into anthocyanin biosynthesis and accumulation in purple potatoes, offering valuable candidate genes for the future breeding of colored potatoes.

## 1. Introduction

Anthocyanin, a significant secondary metabolite in potatoes, is a class of water-soluble natural pigments prevalent in plants, imparting vibrant colors to various plant tissues [1]. Widely utilized in the healthcare industry, anthocyanins exhibit free radical scavenging properties and enhance the organism’s antioxidant capacity. They also aid in reducing cholesterol levels [2], inhibiting tumor cell growth, lowering blood sugar levels, and preventing cancer cell production [3,4,5]. Anthocyanins can improve the ability of plants to resist biotic and abiotic stresses, such as helping plants to resist the attack of diseases and pests, and making plants more resistant to drought, cold, and salinity, thus promoting the growth and development of plants [6,7]. Pigmented potatoes can be used as plant-derived materials for obtaining natural anthocyanins [8]. Colored potatoes hold substantial utilization value and market potential, prompting breeders to develop new varieties with a high anthocyanin content. Over 600 anthocyanin types have been identified in nature [9], with nearly 50 known varieties, including the six most common: geranium pigment (pelargonidin), cornflower pigment (cyanidin), delphinium pigment (delphinidin), paeoniaceae flower pigment (peonidin), petunia flower pigment (petunia), and mallow pigment (malvidin) [10,11]. Consequently, the anthocyanin pigments in colored potatoes are valuable for developing natural pigments, health foods, and natural antioxidants.

Genes involved in anthocyanin biosynthesis fall into two categories: structural genes shared by plants and regulatory genes. Structural genes such as naringenin chalcone synthase (CHS), dihydrokaempferol 4-reductase (DFR), naringenin 3-dioxygenase (F3H), and anthocyanin 3-O glycosidyltransferase (3GT) were cloned from wild potatoes [12]. Transferring the flavonoid-3′,5′-hydroxylase (F3′5′H) gene into potatoes results in purple stems and skins, and red flesh [13]. The *StUFGT* (3GT) gene, cloned from a potato cultivar, is expressed in roots, stems, and petioles, enhancing the anthocyanin content [14,15]. Regulatory genes include the R2R3MYB transcription factors *StMYBA1* and *StMYB113*, which promote anthocyanin accumulation, and the bHLH transcription factors *StbHLH1* and *StJAF13,* key co-regulators of anthocyanin biosynthesis [16]. The R2R3MYB transcription factor AN1 forms a complex with bHLH and WD40 repeat proteins to regulate anthocyanin synthesis, with AN1/*StJAF13* and AN1/*StbHLH1* interactions enhancing anthocyanin accumulation [17]. The WD40 repeat protein gene *StAN11*, cloned in potatoes, increases DFR expression and deepens tuber coloration when transfected into the Desiree variety [18]. Research has suggested that the main regulators of anthocyanin biosynthesis are the encoding transcription factor R2R3-MYB, bHLH, WD40, and the MBW complex (MYB-bHLH-WD40) [19]. *StMYB113* and *StMYB308*, possibly related to anthocyanin synthesis, were screened under low-temperature conditions based on the low-temperature-responsive potato *StMYB* genes family analysis obtained by transcriptome sequencing [20]. *StWRKY70* promotes anthocyanin accumulation by interacting with *StAN1* [21].

In recent years, the integration of high-throughput functional genomics technologies has significantly advanced the study of functional genes regulating tissue development, environmental response, and plant metabolism. Using microarray technology, 27 significantly differentially expressed genes (DEGs) were identified by comparing purple and white potato varieties [22]. Transcriptome analysis of the white ‘New Daping’ and purple ‘Black Beauty’ varieties led to the identification and validation of MYB AN1 and bHLH1 [23]. Kyoungwon established a network mechanism for anthocyanin biosynthesis genes in Hongyoung and Jayoung by correlating 22 metabolites with 119 transcripts [24]. Mechanisms of anthocyanin synthesis in colored potatoes were explored through full-length transcriptome sequencing and a broadly targeted metabolome [25]. The transcriptomics of tuber anthocyanin anabolism were examined by selecting potatoes of different colors during the tuber formation stage [26]. A comparative transcriptome analysis of the wild purple potato and its red mutant was performed using high-throughput RNA sequencing, and 295 DEGs were obtained [27]. Jiao et al. first used a combined transcriptional and metabolic approach to study the flower color mutation in tobacco [28]. Thus, sequencing technology effectively elucidates the mechanisms of potato anthocyanin synthesis, facilitating the identification of significant DEGs and the isolation of key genes involved in anthocyanin anabolism, thereby laying the groundwork for the localization and cloning of genes related to colorful potatoes.

Histological techniques have been extensively employed to analyze anthocyanin biosynthesis in potatoes comprehensively. The expression of each key enzyme gene in the anthocyanin synthesis pathway was detected at the transcriptional level in different colored tubers. Unlike the anthocyanins in aboveground plant organs such as flowers, leaves, and fruits, those in purple potato tubers are synthesized and accumulated without direct light exposure [29]. This characteristic makes potatoes an ideal research material for investigating the mechanism of anthocyanin accumulation in underground organs [30]. In this study, the purple potato line ‘15-12-16’ and the white potato variety ‘Xiazhai 65’ were selected for transcription and metabolism analysis. Transcriptome analysis revealed that *St5GT* plays a key role in pigmentation, with overexpression of *St5GT* in tobacco contributing to anthocyanin accumulation. These results provide a theoretical basis for cloning and utilizing key genes related to anthocyanin biosynthesis and for analyzing molecular regulatory mechanisms.

## 2. Results

### 2.1. Transcriptome Profiling of the Petal Samples of Dark-Purple and White Potatoes

**Figure 1:**
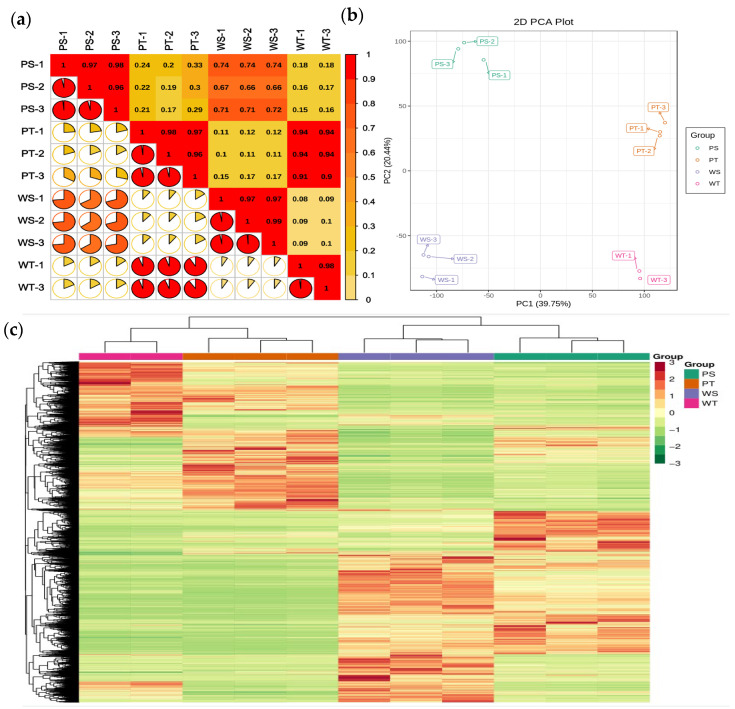
Correlation, heat map, and PCA of the relative differences in anthocyanins in different potato skin and flesh. (**a**) Statistical analysis of inter-sample correlation; (**b**) Heat map visualization. Each sample is visualized in a single column, and a single row represents each metabolite. Red indicates high abundance, green indicates low abundance; (**c**) Score plots for PCA. Purple skin (PS-1, PS-2, PS-3), purple flesh (PT-1, PT-2, PT-3), white skin (WS-1, WS-2, WS-3), white flesh (WT-1, WT-3).

To investigate the transcription factors involved in potato anthocyanin biosynthesis and the underlying molecular regulatory mechanisms, a dark-purple potato line, 15-12-16, and a white potato cultivar, Xiazhai 65, were selected for analysis. Due to the failure of the WT_2 sample, it was discarded. After removing low-quality reads, a total of 494,442,888 clean reads were obtained. The Q30 and GC content percentages ranged from 94.61% to 95.13% and 41.23% to 43.43%, respectively, indicating high-quality transcriptome sequencing data (Table 1). A total of 16,485 genes were functionally annotated in the databases. Principal component analysis (PCA) was used to identify key factors in the transcriptome data, revealing clear distinctions between the different varieties and treatments based on the first two principal components (PC1, PC2). Additionally, a correlation heatmap demonstrated high reproducibility within the biological replicates of the same sample groups (Figure 1). DEG screening criteria were set at |fold change| > 2 and FDR < 0.05, resulting in 5038, 10,634, 6376, and 11,208 DEGs in the PT_vs_WT, PS_vs_PT, PS_vs_WS, and WS_vs_WT comparisons, respectively. PT_vs_WT and PS_vs_WS shared 9148 DEGs, with 2266 genes common to both comparisons.

### 2.2. DEG Function Analysis

**Figure 2:**
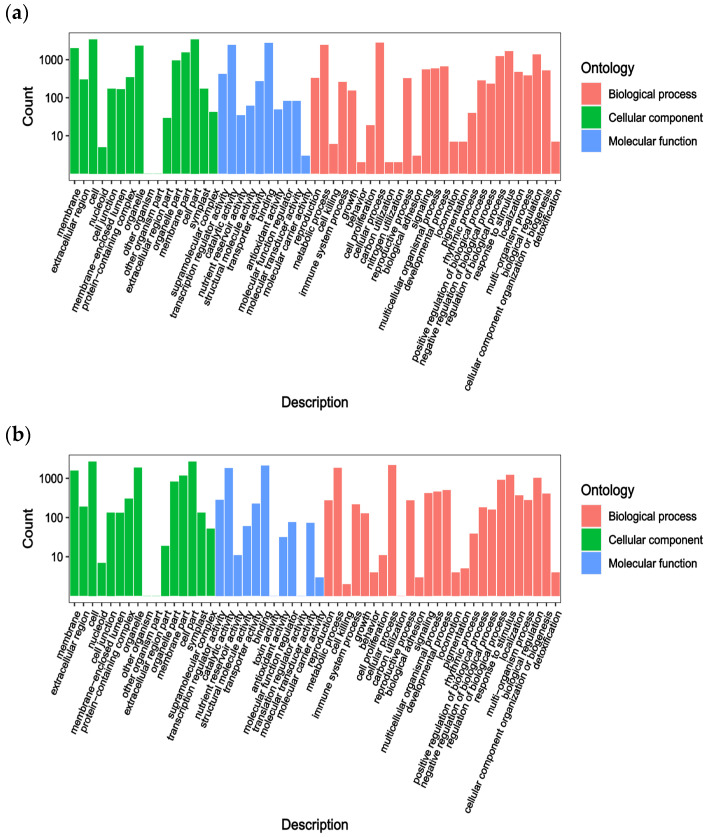
The GO enrichment analysis of DEGs for the (**a**) (PS and WS), (**b**) (PT and WT) groups.

DEGs among the groups are enriched in various GO terms and KEGG pathways. To verify the biological functions of these genes, GO and KEGG enrichment analyses were performed. The transcriptome sequencing results provided GO annotation information for the DEGs, categorizing them into three sections: molecular function, biological process, and cellular composition. Only the 50 GO terms with the lowest *q*-values from each group’s enrichment analysis are presented here (Figure 2). Most DEGs in the PS and WS groups were involved in bacterial defense responses (153), carbohydrate binding (145), regulation of stress responses (141), monooxygenase activity (138), and secondary metabolic processes (137). Notably, 54 and 65 DEGs were annotated in flavonoid biosynthesis and phenylpropanoid biosynthesis pathways related to anthocyanin synthesis (Figure 2a), respectively. In the PT and WT groups, DEGs were enriched in monooxygenase activity (106), UDP-glucosyltransferase activity (98), glucosyltransferase activity (83), secondary active transmembrane transporter protein activity (81), and UDP-glucosyltransferase activity (72) (Figure 2b). Among these, 50 DEGs were annotated in the flavonoid biosynthesis pathway, which is directly linked to anthocyanin biosynthesis.

**Figure 3:**
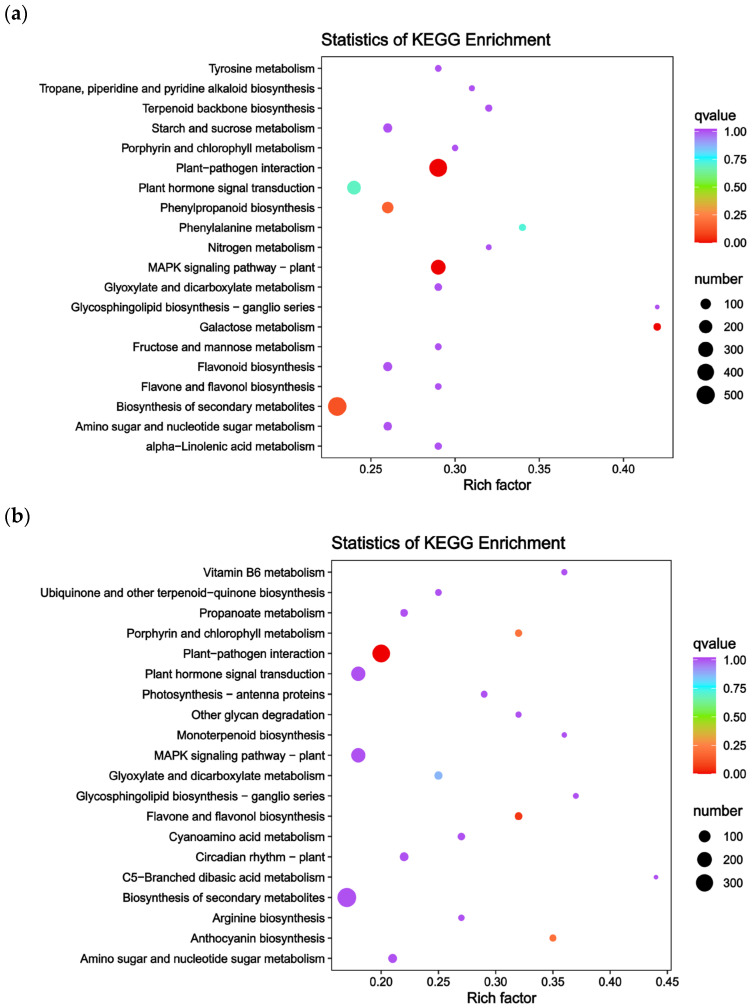
KEGG pathway enrichment map of differential genes for the (**a**) (PS and WS), (**b**) (PT and WT) groups.

To further elucidate the functions of the DEGs, KEGG enrichment analysis was conducted. The analysis revealed that 2078 DEGs in PS and WS were enriched in 130 KEGG pathways, while 5624 genes in PT and WT were enriched in 133 pathways. These pathways included plant–pathogen interaction (Ko04626), phytohormone signaling (Ko04075), and biosynthesis of secondary metabolites (Ko01110), among others. In PS and WS, genes related to anthocyanin synthesis were significantly enriched in secondary metabolite biosynthesis (Ko01110, 515 genes), flavonoid biosynthesis (Ko00940, 135 genes), and phenylpropanoid biosynthesis (Ko00941, 63 genes) (Figure 3a). Similarly, in PT and WT, significant enrichment was observed in the flavonoid biosynthesis (Ko00941, 15 genes) and anthocyanin biosynthesis (Ko00942, 46 genes) pathways (Figure 3b). These findings suggest a strong association between these genes and the formation of purple potato coloration.

### 2.3. Major Transcription Factors Were Differentially Regulated Between Purple and White Potatoes

**Figure 4:**
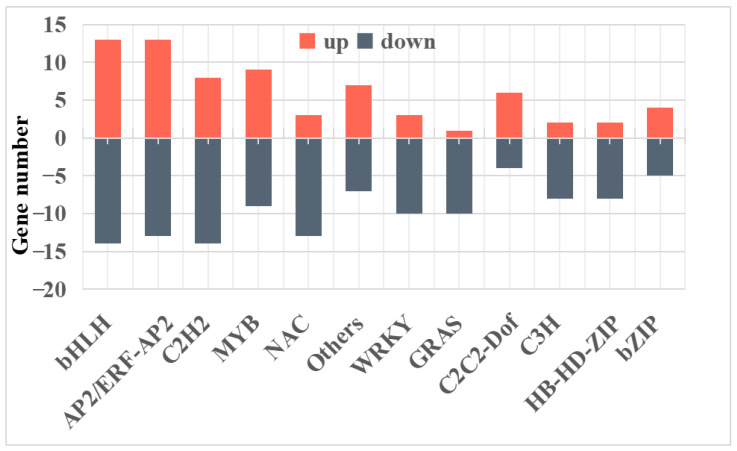
Histogram of the number of genes in the top 12 differentially expressed transcription factor families with the most members.

The primary transcription factor families differentially expressed between PT and WT were analyzed, revealing 344 differentially expressed transcription factors (TFs) across 66 TF families. Among these, the bHLH and AP2/ERF-AP2 families were the most abundant, comprising 27 and 26 members, respectively. The C2H2, MYB, NAC, Others, and WRKY families included 22, 18, 16, 14, and 13 members, respectively. The number and expression levels of the 12 main transcription factors are depicted in Figure 4. Notably, the bHLH, AP2/ERF-AP2, MYB, C2H2, and WRKY families demonstrated more active members involved in gene regulation, suggesting their vital role in regulating structural genes responsible for color formation. Specifically, MYC2 bHLH protein (PGSC0003DMG400000232), tDNA binding protein (PGSC0003DMG400005528) (bHLH), GAMYB-like1 (PGSC0003DMG400022689) (MYB), and MYB-like transcription factor DIVARICATA (PGSC0003DMG400030348) (MYB) exhibited significantly upregulated expression in purple potatoes but low expression in white potatoes. Additionally, Anthocyanin 1 (PGSC0003DMG400012891) (bHLH) and MYB-like DNA-binding protein (PGSC0003DMG400026758) were significantly upregulated in purple potatoes and absent in white potatoes. These results indicate that these genes are significant regulators of purple skin and flesh color formation.

### 2.4. Metabolome Analysis

Metabolomic analysis was conducted to elucidate the anthocyanin types and corresponding levels between white and purple potatoes. Two potato cultivars were planted simultaneously and grown in the same field and under the same conditions, and anthocyanin metabolites of the flesh and skin of the two potatos were analyzed by UPLC-ESI-MS/MS, with identification of anthocyanin fractions using MWDB’s own constructed database. A total of 34 anthocyanin-related compounds were detected in the metabolome. All detected components were quantified and the contents are shown in Table 2, including cyanidins (8), peonidins (4), delphinidins (6), pelargonidins (4), flavonoids (4), malvidins (3), and petunidins (5). Cyanidin and delphinidins were the most abundant, with 8 and 6 compounds, respectively, accounting for 23.53% and 17.65% of the total compounds. Per the histogram of anthocyanidin levels, Petunidin 3-O-rutinoside, Delphinidin 3-O-rutinoside, and Cyanidin 3-O-rutinoside were present at high levels in the purple flesh (PT) but were absent in white potatoes; Petunidin 3-O-rutinoside, Malvidin 3-O-rutinoside, and Peonidin 3-O-rutinoside were present at high levels in the purple skin (PS) but were absent in white potatoes. The results indicate that Petunidin 3-O-rutinoside, Delphinidin 3-O-rutinoside, and Cyanidin 3-O-rutinoside are significantly associated with the formation of purple potato flesh colors, and Petunidin 3-O-rutinoside, Malvidin 3-O-rutinoside, and Peonidin 3-O-rutinoside are significantly associated with the formation of purple potato skin colors, confirming the significant roles of Petunidin 3-O-rutinoside, Delphinidin 3-O-rutinoside, Cyanidin 3-O-rutinoside, Malvidin 3-O-rutinoside, and Peonidin 3-O-rutinoside in purple flesh and skin color formation.

### 2.5. Differential Expression of Structural Genes Related to Anthocyanidin Biosynthetic Pathways in the Potato 15-12-16 and Xiazhai65

**Figure 5:**
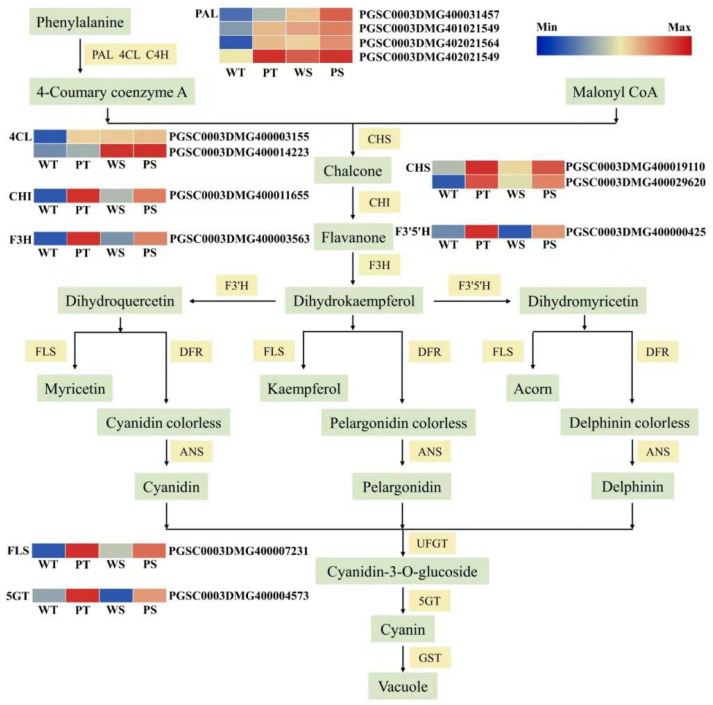
Anthocyanin biosynthetic pathway and their gene-expression levels in potato skin and flesh.

Structural genes in the anthocyanin biosynthesis pathway influence anthocyanin production by encoding relevant enzymes. According to the structural genes differential expression, 13 structural genes were screened to identify critical genes controlling anthocyanin content in potato flesh. These genes included four phenylalanine ammonia-lyase (PAL), two 4-coumarate-CoA ligase (4CL), CHS, and one each of chalcone isomerase (CHI), F3H, F3’5’H, flavonol synthase (FLS), and anthocyanin 5-O glycosidyltransferase (5GT). These genes were significantly upregulated or highly expressed in the purple potato line 15-12-16’ s skin and flesh but were downregulated or absent in the white potato Xiazhai 65 (Figure 5). As critical structural genes in the anthocyanin synthesis pathway, they likely play a role in anthocyanin production in the purple potato line 15-12-16.

### 2.6. Integrated Analysis of the Transcriptome and Metabolome

**Figure 6:**
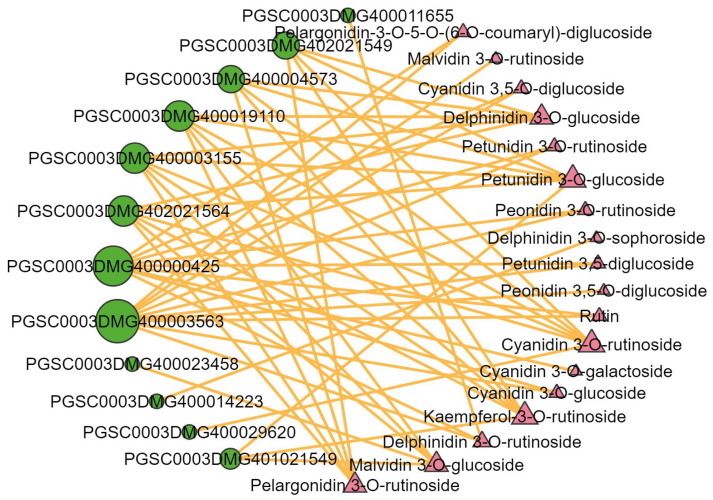
A correlation network between differentially expressed structural genes and metabolites. The green circles represent the differential genes and the pink triangles represent the differential metabolites. The correlations with R > 0.8 and *p* < 0.05 were deemed significant. The orange lines represent positive correlations. The graph’s size represents the degree of correlation.

The correlation network was carried out to further investigate the relationship between the differential expression of structural genes related to anthocyanidin biosynthetic and metabolites. Correlations between the differential expression of genes and metabolites were analyzed using Pearson’s correlation coefficients. Pearson’s correlation analysis showed that 12 DEGs involved in anthocyanin biosynthesis were significantly correlated with 18 metabolites (Figure 6). The 12 structural genes included four PAL genes, two CHS genes, two 4CL genes, one CHI gene, one F3H gene, one F3’5’H gene, one CHS gene, and one 5GT gene. F3H and F3’5’H genes were significantly correlated with 10 and 9 metabolites, respectively. PAL (PGSC0003DMG402021564), 4CL (PGSC0003DMG400003155), and CHS (PGSC0003DMG400019110) were correlated with six identical metabolites, including delphinidin-3-O-glucoside, petunidin-3-O-glucoside, cyanidin-3-O-rutinoside, malvidin-3-O-glucoside, kaempferol-3-O-rutinoside, and pelargonidin-3-O-rutinoside. 5GT (PGSC0003DMG400004573) shares the same five metabolites as the above three genes and lacks one metabolite, malvidin-3-O-glucoside. They are associated with anthocyanin biosynthesis and may be the main structural genes that contribute to the development of purple potatoes.

### 2.7. Validation of the Expression of DEGs by qRT-PCR

**Figure 7:**
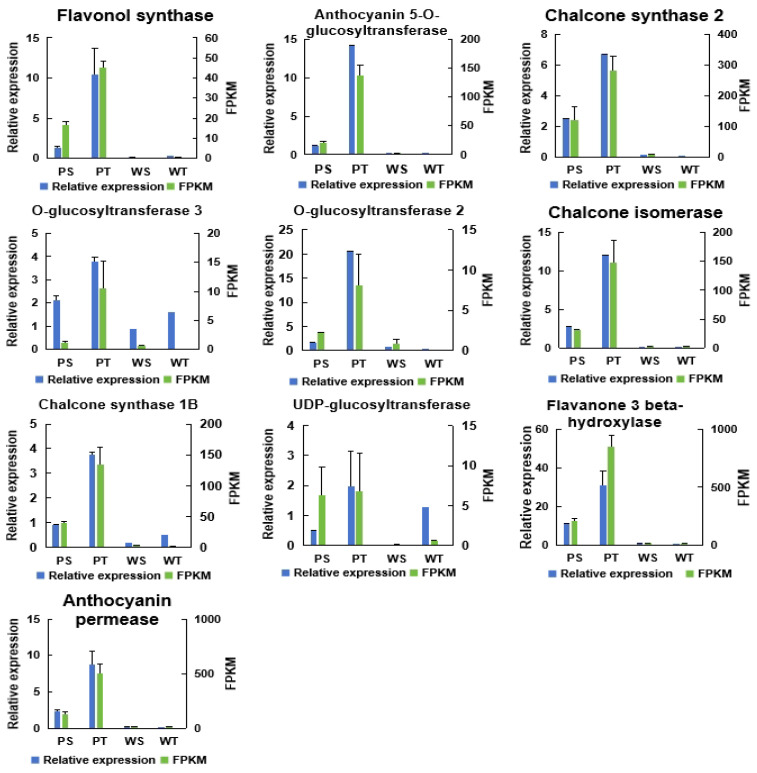
Validation of expression patterns of selected genes by qRT-PCR. Transcript abundances detected by transcriptome sequencing and expressed in FPKM are shown on the right. Relative expression levels detected by RT-qPCR and expressed in 2^–∆∆CT^ are shown on the left. Data are presented as the mean of three replicates with three biological repeats.

Ten DEGs related to anthocyanin synthesis were selected and verified using the qRT-PCR method. The qRT-PCR primers were synthesized by Shanghai Shenggong Bioengineering Co., Ltd. ACTIN (GenBank accession no. X83206) was validated as constitutively expressed and appropriate as an internal control, which served as the reference gene. Gene expression levels were analyzed using the 2^–∆∆CT^ method. The qRT-PCR results were consistent with the RNA-Seq data (Figure 7), confirming the RNA-Seq findings’ reliability.

### 2.8. Heterologous Expression of St5GT in Tobacco

**Figure 8:**
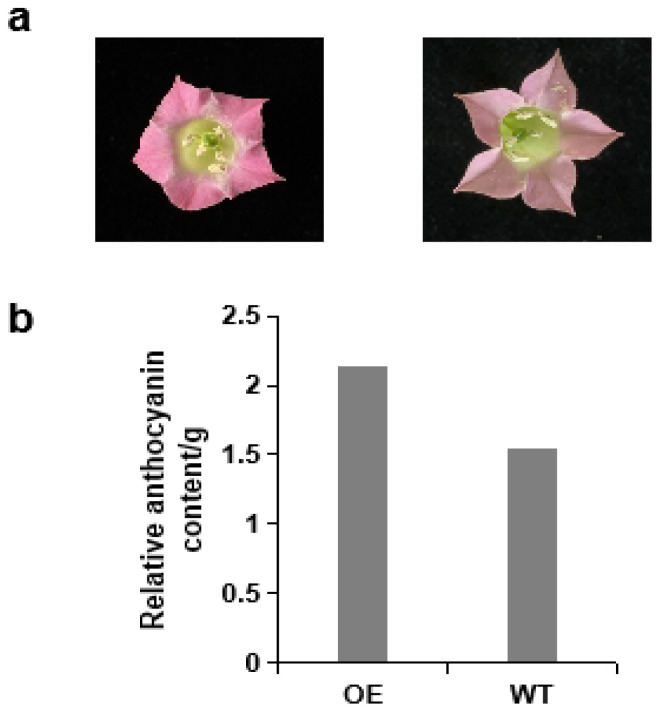
Overexpression of *St5GT* increases anthocyanin accumulation in tobacco. (**a**) On the left is the flower color of tobacco overexpressing *St5GT*, and on the right is the wild-type flower; (**b**) Relative anthocyanin content in overexpressing (OE) and wild-type (WT) plants.

To elucidate the regulation of key genes governing potato coloration, the structural genes involved in anthocyanin biosynthesis were examined for their primary contribution to potato coloring. Among the 13 differentially expressed structural genes in the PT and WT groups, PGSC0003DMG400004573 (*St5GT*) displayed high expression in purple potatoes but was nearly absent in white potatoes. As a result, *St5GT* was selected as a candidate gene for overexpression in tobacco, and an independent overexpression (OE) line was obtained. The color of flowers of the three *St5GT* OE lines significantly turned red compared with the untransformed tobacco (WT). We measured the anthocyanin content of the OE lines and the WT, and the findings revealed that *St5GT* significantly enhances anthocyanin accumulation, with a markedly higher anthocyanin content in the transgenic line (Figure 8). Therefore, *St5GT* was involved in the visible colorations of the potato and is pivotal in the synthesis of potato anthocyanins.

## 3. Discussion

Variations in anthocyanin content and composition result in a diverse range of colors in potato tissues. Most previous studies have analyzed the mechanisms of potato color formation using metabolome and transcriptome methods, identifying key genes and enzymes involved in color formation and anthocyanin accumulation in potatoes [22,23,24,25,26]. However, the regulation of potato color is a complex process. To screen for key genes involved in anthocyanin biosynthesis in purple potatoes, the regulation mechanism of potato color was further studied through metabolite and gene expression analysis based on transcriptome analysis and metabolic profiling.

In this study, integrated analysis of the transcriptional and metabolic results showed that 12 differential expressions of structural genes related to anthocyanidin biosynthetic significantly correlated with 18 metabolites. F3H, F3’5’H, PAL, 4CL, CHS, and 5GT genes are associated with anthocyanin biosynthesis and may be the primary structural genes in developing purple potatoes. F3H catalyzes the conversion of flavanones to dihydroflavonols, serving as an intermediate step in anthocyanin synthesis, while F3’5’H is associated with specific color presentations in plants [31]. In potatoes, F3’5’H plays a key role in determining tuber color, with its expression level influencing whether tubers appear red or purple [32]. The F3H gene contributes to anthocyanin accumulation in the arabidopsis tt7 mutant and tobacco [33,34]. Overexpression of *Fh5GT* in tobacco resulted in enhanced-red pigmentation in transgenic tobacco [35]. *StbHLH1* and *StJAF13*, which interact with MYB transcription factors, were found to be key co-regulated genes for tuber anthocyanin synthesis, and *StMYB44*, a transcription factor whose expression is induced at high temperature, negatively regulated tuber anthocyanin synthesis [16]. The transcription factors *StMYB113* and *StMYB308* were found to promote tuber anthocyanin synthesis in response to low-temperature induction [20]. Also, the identification of a novel WRKY transcription factor, *StWRKY70*, was found to be involved in the regulation of flesh color formation [21]. In our study, *St5GT* was significantly upregulated in purple potatoes but absent in white potatoes. Overexpression of the *St5GT* gene in tobacco revealed that the transgenic lines had significantly darker flower colors compared to the control, and anthocyanin content in the flowers of the transgenic lines was higher than in the control. Anthocyanin 5-O glycosidyltransferase (5GT) is a functioning enzyme that requires glycosylation modifications after the synthesis of anthocyanins at the later stages of anthocyanin biosynthesis, and it uses UDPG as a glycosyl carrier for the 5-O glycosidization of anthocyanins. These glycosylation modifications can make anthocyanins more stable and increase the color change of anthocyanins, and the ectopic expression of 5GT also improves plant defenses against pathogen infections [36,37,38]. The findings further revealed that the structural gene *St5GT* involved in anthocyanin synthesis, is a critical gene in the synthesis of potato anthocyanins, which provides gene resources for the later development of purple potato breeding, and also lays the foundation for the effective utilization of purple potato 15-12-16 in purple potato breeding.

Transcription factors regulate gene expression [39]. The most common TFs include the ternary-protein complex (MBW), composed of DNA-binding R2R3-MYB transcription factor (MYB), basic helix-loop-helix (bHLH) transcription factor, and WD40 repeat protein (WD40) [23,40,41]. Previous studies have demonstrated that MYB alone, coexpression of MYB and bHLH, and formation of the MYB-bHLH-WD40 complex can all induce anthocyanin accumulation in plants [42,43,44,45]. MYB transcription factors are particularly effective in promoting anthocyanin accumulation [44,46,47,48,49,50,51]. Both R2R3MYB-*StAN1* and *StbHLH1* influence the pigmentation of potato skin and flesh [52]. Additionally, TFs such as AP2/ERF [53], the WRKY family [54,55], and the bZIP family [56,57] underscore the significance of transcriptional regulation in controlling flavonoid biosynthesis [58]. In this study, the bHLH, AP2/ERF-AP2, MYB, C2H2, and WRKY TF families exhibited significant involvement in gene regulation. Notably, Anthocyanin 1 (PGSC0003DMG400012891) (bHLH) and MYB-like DNA-binding protein (PGSC0003DMG400026758) were significantly upregulated in purple potatoes but absent in white potatoes, suggesting these genes are crucial for regulating structural genes involved in potato coloration.

Previous research has isolated and cloned several structural and regulatory genes related to anthocyanin biosynthesis and metabolism in potatoes, investigating their expression and function [13,16,18,59,60]. Utilizing transcriptional and metabolic data, the expression of key genes in the anthocyanin synthesis pathway was screened in different colored potato skins and flesh at both transcriptional and metabolic levels. This study identified genes related to anthocyanin synthesis in potatoes, providing resources and technical support for further research on anthocyanin synthesis and accumulation, and accelerating the breeding of new potato varieties.

## 4. Materials and Methods

### 4.1. Plant Material

**Figure 9:**
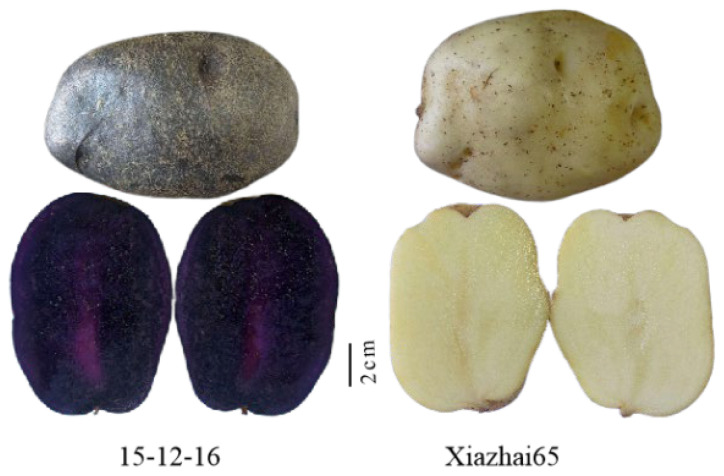
The phenotypic characters of 15-12-16 and Xiazhai 65. Among them, 15-12-16 is a potato variety with purple skin and purple flesh, and Xiazhai 65 is a potato variety with white skin and white flesh.

A dark-purple potato cultivar, 15-12-16 (dark-purple skin and purple flesh), and a white potato cultivar, ‘Xiazhai 65’ (white skin and white flesh), were selected in this study (Figure 9). Samples from these two cultivars were collected when fresh tubers were harvested and then cleaned with sterilized water. Skin tissue was meticulously separated from cortex tissue using a scalpel to minimize flesh contamination. Each treatment had three biological replicates, designated as follows: purple skin (PS_1, PS_2, PS_3), purple flesh (PT_1, PT_2, PT_3), white skin (WS_1, WS_2, WS_3), and white flesh (WT_1, WT_2, WT_3). The skin and flesh of the potatoes were immediately frozen in liquid nitrogen and stored at −80 °C until further analysis.

### 4.2. RNA Extraction, Library Construction, and Sequencing

RNA was extracted from the potato skin and flesh of ‘15-12-16’ (PS for skin and PT for flesh) and ‘Xiazhai 65’ (WS for skin and WT for flesh). The freeze-dried samples were pulverized using a mixer mill (MM 400, Retsch, Handelson, Hamburg, Germany) with a zirconia bead for 1.5 min at 30 Hz. Fifty milligrams of the resulting powder was extracted with 0.5 mL methanol/water/hydrochloric acid (799:200:1, *v*/*v*/*v*). The integrity and contamination of the RNA were assessed using agarose gel electrophoresis and an Agilent 2100 bioanalyzer (Agilent, Palo Alto, CA, USA). RNA concentration was measured with a Qubit 2.0 fluorometer (Invitrogen, Carlsbad, CA, USA). RNA samples that passed the quality check were used to construct cDNA libraries, which were sequenced on the Illumina HiSeq platform (MetWare Metabolism) (Metware Biotechnology Co., Ltd., Wuhan, China). The method of RNA extraction, quantification and transcriptome sequencing were performed as fully described by Lu et al. [61]. The libraries were sequenced on an Illumina Hiseq platform and 150 bp paired-end reads were generated.

### 4.3. RNA-Seq Analysis

The reference genome of the doubled haploid *S. tuberosum* group Phureja clone DM1-3 516R44 (referred to as DM) was downloaded from the ENSEMBL plant database (ftp://ftp.ensemblgenomes.org/pub/plants/release-34/fasta/solanum_tuberosum/dna/ (accessed on 28 December 2020)) [62]. Differential expression analysis was conducted using DESeq2 [63,64], and gene read counts were screened for DEGs using feature Counts [65]. DEGs were identified based on criteria of |log_2_Fold Change| ≥ 1 and FDR < 0.05. All DEGs were annotated into GO and KEGG databases, and the distribution of DEGs, functional classifications, and significantly enriched pathways were analyzed. Plant TFs were predicted with iTAK software 1.7a, the TFs were identified and classified according to the method as described by Perez-Rodriguez et al. [66]. The hmmscan program was used to identify TFs among the DEGs [67].

### 4.4. The Method of Metabolome Analysis

The sample preparation, analysis, and metabolite quantification were conducted by MWDB (Metware Biotechnology Co., Ltd., Wuhan, China) following their standard procedures [68,69,70] using a UPLC-ESI-MS/MS system (UPLC: ExionLC™ AD, https://sciex.com.cn/ (accessed on 17 December 2020); MS: Applied Biosystems 6500 Triple Quadrupole, https://sciex.com.cn/). Identification of anthocyanin fractions using MWDB’s own constructed database. The standard curves of thirty-four anthocyanin components (Cya-3,5-O-diglu, Cya-3-O-ara, Cya-3-O-gal, Cya-3-O-glu, Cya-3-O-rut, Cya-3-O-sop, Cya-3-O-(coumaryl)-glu, Cya-3-O-xyl, Del-3-O-ara, Del-3-O-glu, Del-3-O-rut, Del-3-O-sop, Del-3-O-(6-O-acetyl)-glu, Del-3-O-(coumaryl)-glu, Fla_dihydromyricetin, Fla_kaempferol-3-O-rut, Mal-3,5-O-diglu, Mal-3-O-glu, Mal-3-O-rut, Pel-3-O-glu, Pel-3-O-rut, Pel-3-O-sam, Pel-3-O-5-O-(6-O-coumaryl)-diglu, Peo-3-O-glu, Peo-3,5-O-diglu, Peo-3-O-rut, Peo-3-(caf-glucosyl-glu)-5-glu, Pet-3,5-O-diglu, Pet-3-O-ara, Pet-3-O-glu, Pet-3-O-rut, Pet-3-O-sop, Fla_quercetin-glu, Fla_rutin) were plotted and linear equations of the standard curves were calculated. Thus, the anthocyanin component content was calculated accurately.

### 4.5. Combined Transcriptome and Metabolome Analysis

The obtained differentially expressed structural genes involved in anthocyanin biosynthesis were mapped in the anthocyanin biosynthesis pathway. The FPKM values of the structural genes involved in the pathway were standardized and plotted into heat maps to the pathway map. Combined with transcriptomic differential genes and metabolites, they were selected for correlation network diagram analysis.

### 4.6. Expression Validation by Quantitative Real-Time PCR Analysis

Based on the CDS sequences of DEGs obtained from NCBI, the potato Actin gene (GenBank accession no. X83206) was used as the reference gene. Specific primers in Table S1 were designed and synthesized, and RNA reverse transcription from the skin and flesh of potato cultivars ‘15-12-16’ and ‘Xiazhai 65’ produced the cDNA. This cDNA was employed as a template for real-time fluorescence quantitative analysis. PCR was conducted in a 20 µL reaction mixture comprising 10 µL TB Green^®^ Premix Ex TaqTM II, 0.4 µL of each primer, 7.2 µL double-distilled H_2_O, and 2 µL cDNA. The PCR program was: 95 °C for 30 s; 95 °C for 10 s, 60 °C for 32 s, for 40 cycles; 95 °C for 60 s; 60 °C for 60 s; and 55 °C for 10 s. Each sample underwent triplicate analysis, and the data were processed to calculate relative expression using the 2^−ΔΔCt^ method [71].

### 4.7. Tobacco Transformation Assays

*St5GT* (PGSC0003DMG400004573) was cloned into the pJAM1502-*St5GT* vector and subsequently transformed into Agrobacterium tumefaciens GV3101. The leaf discs method was employed for the genetic transformation of tobacco, and the resulting transgenic lines were transplanted into pots to observe plant phenology. Samples were frozen in liquid nitrogen and stored at −80 °C for anthocyanin content determination. RNA extraction and reverse transcription were performed using the Tengen RNA Extraction Kit and the PrimeScriptTM RT Reagent Kit with gDNA Eraser (TaKaRa, Dalian, China). The primers used are listed in Table 3.

### 4.8. Detection of Anthocyanins

The total anthocyanin content of tobacco plants was determined using a modified ethanol hydrochloride ultrasonic extraction method [72]. Fresh tobacco samples were ground into powder using a mortar and pestle with liquid nitrogen. Forty milligrams of the powdered sample were transferred into a centrifuge tube containing the extraction solution at a material-to-liquid ratio of 1 g: 10 mL. The samples were ultrasonically extracted at 45 °C for 30 min and then centrifuged at 10,000*g* × rpm for 10 min at room temperature. The supernatant was collected, and the absorbance values of the anthocyanin extract at 530 nm and 657 nm were measured using a UV spectrophotometer (Beijing Purkinje General Instrument Co., Ltd., Beijing, China). The anthocyanin content was calculated using the formula *Q*_Anthocyanins_ = (*A*_530_ − 0.25 × *A*_657_) × *M*^−1^, where Q_Anthocyanins_ represents the anthocyanin amount, A_530_ and A_657_ are the absorbances at the respective wavelengths, and M (g) is the weight of the plant material used for extraction [73].

## 5. Conclusions

In this study, we used the transcriptional and metabolic data of the high-anthocyanin content purple-skin and purple-flesh potato line 15-12-16 and the white-skin and white-flesh potato variety Xiazhai 65. We screened and detected the expression of the critical enzyme genes of the anthocyanin synthesis pathway in different colors of potato skins and tubers. Integrated analysis of the transcriptional and metabolic results showed that 12 differential expressions of structural genes related to anthocyanidin biosynthetic significantly correlated with 18 anthocyanin metabolites. The expression of ten differentially expressed genes (DEGs) was validated through quantitative real-time PCR. Among these genes, *St5GT* was significantly upregulated in purple potatoes but absent in white potatoes. Overexpression of the *St5GT* gene in tobacco can increase the anthocyanin content in the flowers of the transgenic lines compared to the control, so the *St5GT* gene is involved in anthocyanin synthesis and is a key gene in the synthesis of potato anthocyanins. In conclusion, these findings provide new insights into the anthocyanin biosynthesis and accumulation in purple potatoes, offering valuable candidate genes for the future breeding of colored potatoes.

## Figures and Tables

**Table 1:** The data quality analysis in the investigated samples.

Sample	Raw Reads	Clean Reads	Clean Base (G)	Error Rate (%)	Q20 (%)	Q30 (%)	GC Content (%)
PS-1	48860478	47610640	7.14	0.02	98.27	94.65	42.18
PS-2	45029580	44036178	6.61	0.02	98.27	94.7	42.47
PS-3	55048496	53272348	7.99	0.02	98.23	94.61	43.43
PT-1	43451020	41554096	6.23	0.02	98.32	94.78	42.8
PT-2	46305534	44798410	6.72	0.02	98.51	95.13	42.18
PT-3	44340698	42478042	6.37	0.02	98.36	94.84	42.02
WS-1	45139190	43512134	6.53	0.02	98.29	94.64	41.23
WS-2	50208396	48873826	7.33	0.02	98.38	94.91	41.45
WS-3	43767374	41440758	6.22	0.02	98.35	94.91	41.59
WT-1	43302236	41430580	6.21	0.02	98.4	95.01	42.09
WT-3	46903456	45435876	6.82	0.02	98.38	94.93	42.15

**Table 2:** All detected anthocyanin metabolites and their contents between purple and white potatoes.

Compounds	RT/min	M/Z	PS	PT	WS	WT
Cyanidin 3,5-O-diglucoside	4.28	611.2	0.0168 ± 0.0013 ^a^	0.0162 ± 0.0015 ^a^	N/A	N/A
Cyanidin 3-O-arabinoside	6.17	419.1	N/A	0.0024 ± 0.0012 ^a^	N/A	N/A
Cyanidin 3-O-galactoside	5.18	449.1	0.0259 ± 0.0033 ^a^	0.0276 ± 0.0059 ^a^	0.0130 ± 0.0089 ^a^	0.0151 ± 0.0105 ^a^
Cyanidin 3-O-glucoside	5.75	449.1	0.0061 ± 0.0017 ^b^	0.0800 ± 0.0087 ^a^	0.0054 ± 0.0044 ^b^	N/A
Cyanidin 3-O-rutinoside	6.36	595.17	0.5193 ± 0.2638 ^b^	5.4433 ± 0.9150 ^a^	0.0068 ± 0.0097 ^b^	N/A
Cyanidin 3-O-sophoroside	5.15	611.2	N/A	0.0101 ± 0.0020 ^a^	N/A	N/A
Cyanidin-3-O-(coumaryl)-glucoside	11.24	595.15	N/A	0.0056 ± 0.0013 ^a^	N/A	N/A
Cyanidin-3-O-xyloside	7.9	419.1	N/A	0.0002 ± 0.0001 ^a^	N/A	N/A
Peonidin 3-O-glucoside	7.44	463.3	0.0669 ± 0.0065 ^a^	0.0073 ± 0.0032 ^c^	0.0337 ± 0.0119 ^b^	N/A
Peonidin 3,5-O-diglucoside	5.66	625.2	0.1307 ± 0.0240 ^a^	0.0325 ± 0.0024 ^b^	N/A	N/A
Peonidin 3-O-rutinoside	7.98	609.5	0.6447 ± 0.2984 ^a^	0.7067 ± 0.0379 ^a^	N/A	N/A
Peonidin-3-(caffeoyl-glucosylglucoside)-5-glucoside	10.25	949.26	0.0119 ± 0.0012 ^a^	N/A	N/A	N/A
Delphinidin 3-O-arabinoside	5.26	435.5	N/A	0.0007 ± 0.0005 ^a^	N/A	N/A
Delphinidin 3-O-glucoside	4.73	465.1	0.0447 ± 0.0204 ^b^	1.2633 ± 0.0379 ^a^	0.0360 ± 0.0330 ^b^	N/A
Delphinidin 3-O-rutinoside	5.3	611.1	0.0865 ± 0.0126 ^b^	10.9133 ± 0.9801 ^a^	N/A	N/A
Delphinidin 3-O-sophoroside	4.34	627.15	0.0089 ± 0.0012 ^b^	N/A	0.0177 ± 0.0026 ^a^	N/A
Delphinidin-3-O-(6-O-acetyl)-glucoside	9.02	507.11	0.0386 ± 0.0228 ^a^	N/A	N/A	N/A
Delphinidin-3-O-(coumaryl)-glucoside	10.43	611.14	N/A	0.0109 ± 0.0032 ^a^	N/A	N/A
Pelargonidin 3-O-glucoside	6.72	433.2	N/A	0.0065 ± 0.0012 ^a^	N/A	N/A
Pelargonidin 3-O-rutinoside	7.39	579.06	0.1957 ± 0.0885 ^b^	0.9173 ± 0.0386 ^a^	0.0210 ± 0.0254 ^c^	N/A
Pelargonidin 3-O-sambubioside	6.98	565.2	N/A	0.0033 ± 0.0008 ^a^	N/A	N/A
Pelargonidin-3-O-5-O-(6-O-coumaryl)-diglucoside	11.05	741.2	0.0040 ± 0.0032 ^b^	0.0160 ± 0.0025 ^a^	N/A	N/A
Quercetin 3-O-glucoside	8.91	465.1	0.2983 ± 0.0480 ^a^	N/A	N/A	0.0073 ± 0.0018 ^b^
Rutin	8.97	611.2	0.4180 ± 0.1113 ^a^	0.3663 ± 0.1190 ^a^	N/A	N/A
Dihydromyricetin	3.6	321.1	N/A	0.1697 ± 0.0091 ^a^	N/A	N/A
Kaempferol-3-O-rutinoside	10.67	595.2	0.6143 ± 0.0593 ^a^	0.1647 ± 0.0471 ^b^	0.0327 ± 0.0390 ^c^	N/A
Malvidin 3,5-diglucoside	6.02	655.4	0.4383 ± 0.1222 ^a^	0.0245 ± 0.0081 ^b^	N/A	N/A
Malvidin 3-O-glucoside	7.96	493.1	0.9527 ± 0.7722 ^a^	0.0099 ± 0.0054 ^a^	0.5053 ± 0.4425 ^a^	N/A
Malvidin 3-O-rutinoside	8.39	639.06	0.7993 ± 0.3656 ^a^	0.2690 ± 0.0193 ^b^	N/A	N/A
Petunidin 3,5-diglucoside	4.82	641.2	0.4970 ± 0.1206 ^a^	0.5580 ± 0.0520 ^a^	N/A	N/A
Petunidin 3-O-arabinoside	6.99	449.1	N/A	0.0060 ± 0.0024 ^a^	N/A	N/A
Petunidin 3-O-glucoside	6.5	479.1	0.1437 ± 0.0244 ^a^	0.0793 ± 0.0155 ^b^	0.0323 ± 0.0339 ^bc^	N/A
Petunidin 3-O-rutinoside	7	625.06	1.9733 ± 0.3050 ^b^	15.1333 ± 0.5132 ^a^	N/A	N/A
Petunidin 3-O-sophoroside	5.88	641.11	N/A	0.4613 ± 0.0268 ^a^	N/A	N/A

N/A: not detected. Lowercase letters (a, b, c) indicate a significant level of anthocyanin components in different samples.

**Table 3:** Primers used in this study.

Gene ID	Forward Primer (5′-3′)	Reverse Primer (5′-3′)
PGSC0003DMG400004573 *qSt5GT*	TGGATGGAGGGGTAAAAGGGGAAG	ACCTTCTTTCACAGCTTCTCTAGCC
PGSC0003DMG400004573 attB-*St5GT*	AAAAAGCAGGCTTCATGGTGAAGCCTCATGTTAT	AGAAAGCTGGGTCTCAATAACCTTTGGCAATTTCTT
GenBank No. X83206	AGATGCTTACGCTGGATGGAATGC	TTCCGGTGTGGTTGGATTCTGTTC

## Data Availability

The RNA-Seq data generated for the analysis are deposited at the Chinese Academy of Sciences (GSA: CRA017703).

## Supplementary Materials

The following supporting information can be downloaded at: https://www.mdpi.com/article/10.3390/ijms252312884/s1.
