# Supplementary material for: Combined Metabolome and Transcriptome Analyses Reveals Anthocyanin Biosynthesis Profiles Between Purple and White Potatoes
Source: Int J Mol Sci. 2024 Nov 29;25(23):12884. doi: 10.3390/ijms252312884 (PMC11641821; doi:10.3390/ijms252312884)
Supplement: Supplementary file 1 [file ijms-25-12884-s001.zip › ijms-3268666-supplementary.pdf]

Table S1. The primers information used in qRT-PCR analysis in this study.

Gene ID	Forward Primer (5′-3′)	Reverse Primer (5′-3′)	amplified fragment length	annealing temperature
Chalcone synthase 2	GGCTGACTACCAA CTCACTAAG	TAACAGTCCCAC CAGCAAAG	95bp	60°C
Chalcone synthase 1B	AGGACTTGGCTGA GAACAAC	TTGGCCTACCAGG CTATCTA	113bp	60°C
O-glucosyltransfe rase 3	GATTGGAGTCCCA CAGTTATCC	CCTTCTTCTTCTAC TCTTGCTCTC	96bp	60°C
O-glucosyltransfe rase 2	TGTTACACACTGT GGATGGAAT	TGGCATTTGTTGT TTGGTCAG	98bp	60°C
UDP-glucosyltran sferase	TGGGACTTGAACT TACTGGTTATC	CCTTTCCCTTGTG TTCTCTCTT	115bp	60°C
Anthocyanin permease	GGGACTTTGGTTA GGGATGATAG	AACCTCCTTGTTC CAGTTAGTC	91bp	60°C
Flavonol synthase	GGCGTAAGTCCTC ATTCTGATAC	GGGCTTTATAGGC ACCCAAATA	108bp	60°C
Flavanone 3 beta-hydroxylase	TGTCTGGTGGCAA GAAAGG	GTCTCTAGCTCGA ATTGGGTATG	113bp	60°C
Anthocyanin 5-O-glucosyltrans ferase	CTCTGGAGAGCTT AGCTTGTG	CTCCACTCTTCCA CACATCTTC	102bp	60°C
Chalcone isomerase	CAGTATGGTGTGC AGCTAGAG	CGAGTGCTTCTTC CTCATCTT	85bp	60°C
